# The risk assessment of rockburst intensity in the highway tunnel based on the variable fuzzy sets theory

**DOI:** 10.1038/s41598-022-27058-1

**Published:** 2023-03-23

**Authors:** Ai-Feng Wang, Xiu-Tao Yang, Xin-Bao Gu

**Affiliations:** 1 School of Architecture, Nanyang Institute of Technology, Nanyang, 473004 Henan China; 2 Henan Planning Design & Research Institute Co. Ltd., Zhengzhou, 450000 Henan China; 3 School of Civil Engineering, Nanyang Institute of Technology, Nanyang, 473004 Henan China

**Keywords:** Natural hazards, Engineering

## Abstract

Rockbursts have important influences on construction safety, so the risk assessment of rockburst intensity has great significance. Firstly, the depth of the rockburst, the uniaxial compressive strength, the stress concentration coefficients, the brittleness coefficients, and the elastic energy index are selected as the evaluation index. Secondly, an assessment model is developed based on the fuzzy variable theory. And the model is proposed to assess the rockburst intensity in the highway tunnel. Finally, the results demonstrate that the results derived from the proposed model are consistent with the current specifications; the accurate rate comes to 100%. The method can determine the risk level of rockburst intensity and provide an alternative scheme. Hence, the study can accurately present a new approach to assess the rockburst intensity in the future.

## Introduction

**Figure 1:**
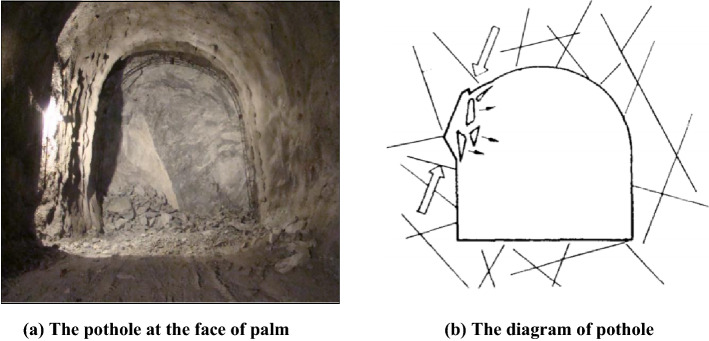
The rockburst in certain tunnel.

The rockburst is defined as the stress concentration phenomenon originating from the disturbance of rock mass in the construction process^1,2^. When the stress concentration arrives at a certain degree, the accumulated elastic strain energy can be released at the instance^3,4^, and then dynamic destabilization occurred. The tunnel's state after the rockburst is plotted in Fig. 1.

The occurrence of rock bursts is characterized by high frequency. According to the relevant statistics^5^, the magnitude of the rockburst cases has arrived more than 1000 times globally. In China, the rockburst hazards have aroused significant loss in underground engineering. For example, in the construction process of the Mount Erlang tunnel and Taipingyi water diversion tunnel, the occurrence magnitudes of rockburst hazards respectively arrive several hundred times^6^, the site construction was stopped frequently, and lots of equipment and staff were damaged. Especially in 8.15, 2016, the occurrence of a rockburst in Liangbaosi Coal Mine, Jining, Shandong Province, resulted in the loss of many life^7^. So predicting and estimating the risk level of rockburst intensity has tremendous significance for the safe construction of the tunnel^8^.

Many researchers in many countries have provided many methods to assess the rockburst intensity^9,10^ in recent years. For example, the fuzzy comprehensive evaluation method^11^, efficacy coefficient method^12^, distance discriminant method^13^, ideal point method^4^, ideal solution sort method^14^, Neural network method^15^, unascertained measure theory^16,17^ and standard cloud theory. They are respectively applied to predict the rockburst intensity. Besides, with the development and popularity of artificial intelligence, machine learning algorithms have been used to predict rockburst hazards. For example, Zhou et al.^18^ established the prediction model of rockburst intensity based on the CRITIC-XGB algorithm; the prediction model of rockburst intensity based on the XG-Boost algorithm for cross-validation is established by Zhang et al.^19^. Liu^20^ adopts the Multidimensional cloud model to test the rationality of weight fusion. The deep neural network model (Adam-DNN) based on the Adam algorithm is suggested by Liang et al.^21^ to predict the rockburst hazards accurately and reliably. The PCA-OPF rockburst prediction model was established by Zhao et al.^22^ based on the principal component analysis in combination with the optimal path forest method. An Intuitionistic Fuzzy Sets-TOPSIS Model is applied to assess the risk level of the rockburst intensity in a Hydraulic Tunnel by Gu et al.^23^

Although the above method has promoted the development of the assessment theory of rockburst intensity, there still needs improvement. For example, the calculative process is complex, and the assessment process in many methods is often quantitative or qualitative. To overcome the shortcomings of the above methods, the entropy weight-variable fuzzy sets are introduced to assess the risk level of rockburst intensity in the tunnel. For example, Yu et al.^24^ applied the VFS method to analyze the classified prediction of rockburst; Wang^25^ use VFS model in combination with the SPA method for the prediction of rockburst; recently, an improved variable fuzzy sets approach is used to predict the rockburst intensity by Wang et al.^26^. In the manuscript, the entropy-VFS model are applied to predict the rockburst in the highway tunnel at first, surrounding rocks in the highway tunnel are very hard, it is a great trial for the prediction of the rockburst intensity in the highway tunnel. The model has many virtues, such as the preciseness of algorithms and operability in practice. It can solve the grading standards and interval form, dramatically improving the traditional fuzzy sets model.

The paper is organized as follows: in “Introduction” section, the engineering overview is introduced at first; in “Methodology” section, theory and methodology based on the entropy-weight variable fuzzy sets model is presented; in “The application of assessment model” section, the assessment model of the rockburst intensity is established, and the assessment results of the proposed model are compared; in “Discussion” section, discussions and comparative analysis are performed; in “Conclusions” section, conclusions are drawn.

## Methodology

### The basic principle

Assume that ${\mathbf{F}}$ belongs to the domain **U**. At any $u \in {\mathbf{U}}$, the number $\mu_{F}^{0} \left( {\text{u}} \right)$ can be determined in the closed interval. The absolute membership relationship is defined as the relation between ${\mathbf{U}}$ and ${\mathbf{F}}$, which can be expressed as^27^:

1 $$\begin{aligned} &\mu_{F}^{0} :U \to \left[ {0,1} \right] \hfill \\ &{\text{u}}\left| \to \right.\mu_{F}^{0} \hfill \\ \end{aligned}$$

In the domain ${\mathbf{U}}$, $u \in {\mathbf{U}}$, there are two opposite fuzzy numbers: ${\mathbf{F}}$ and ${\mathbf{F}}^{c}$. For any variable $u$, there are two determined numbers, $\mu_{F} \left( {\text{u}} \right)$ and $\mu_{{F^{{\text{c}}} }} \left( {\text{u}} \right)$, and the relative membership degree of $u$ to ${\mathbf{F}}$ and ${\mathbf{F}}^{{\text{c}}}$ is defined as:

2 $$\begin{aligned} & \mu_{F} ,\mu_{{F^{c} }} :U \to \left[ {0,1} \right] \\ & {\text{u}}\left| \to \right.\mu_{F} \left( u \right),\mu_{{F^{C} }} \left( u \right) \in \left[ {0,1} \right] \\ \end{aligned}$$

**Figure 2:**
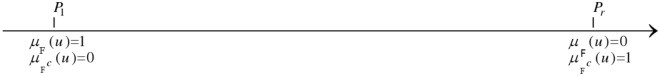
Dynamic change diagram.

Figure 2 depicts the dynamic variable of any number in any closed interval as follows.

**Figure 3:**
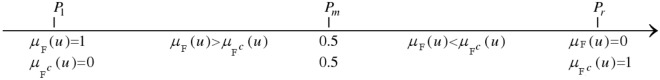
Diagram of opposite fuzzy sets.

The relative membership degree of ${\mathbf{F}}$ and ${\mathbf{F}}^{{\text{c}}}$ meet with $\mu_{F} \left( {\text{u}} \right) + \mu_{{F^{c} }}^{{}} = 1$, $0 \le \mu_{F} \left( {\text{u}} \right) \le 1$, $0 \le \mu_{{F^{{\text{c}}} }} \left( {\text{u}} \right) \le 1$, and they can be expressed as:

3 $$\mathop F\limits_{\sim } = \left\{ {{\text{u,}}\,\mu_{F} \left( {\text{u}} \right),\mu_{{F^{C} }} \left( u \right)\left| {u \in U} \right.} \right\}$$

where $\mathop {\mathbf{F}}\limits_{\sim }$ is the opposite fuzzy set. Figure 3 shows its definition.

The attractive and repelled sets $\mu_{F} \left( {\text{u}} \right)$ and $\mu_{{F^{{\text{c}}} }} \left( {\text{u}} \right)$ can likewise be defined as:

4 $$D_{F} \left( u \right) = \mu_{F} \left( u \right) - \mu_{{F^{c} }}^{{}} \left( u \right)$$

When $\mu_{F} \left( {\text{u}} \right) > \mu_{{F^{{\text{c}}} }}^{{}} \left( u \right)$, $0 \le D_{F} \left( {\text{u}} \right) \le 1$; and when $\mu_{F} \left( {\text{u}} \right) = \mu_{{F^{{\text{c}}} }}$, $D_{F} \left( {\text{u}} \right) = 0$; but when $\mu_{F} \left( {\text{u}} \right) < \mu_{{F^{{\text{c}}} }}^{{}} \left( u \right)$, $- 1 \le D_{F} \left( {\text{u}} \right) \le 0$. The mapping of relative difference function $D_{F} \left( {\text{u}} \right)$ can be expressed as:

5 $$\begin{gathered} D:U \to \left[ {0,1} \right] \hfill \\ {\text{u}}\left| \to \right.D_{F} \left( u \right) \in \left[ { - 1,1} \right] \hfill \\ \end{gathered}$$

**Figure 4:**
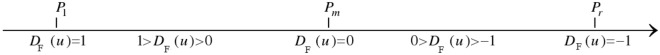
Diagram of relative difference function.

Figure 4 shows the relative difference function of $u$ to ${\mathbf{F}}$.

### Determining the relative membership degree

$X$ is a sample set, which is expressed as:

6 $$X = \left( {x_{ij} } \right)$$

where $X_{ij}$ is the eigenvalue of the index ${\varvec{i}}$ of sample $j$, $i = {1,}2,...,m;j = 1,2,...c$. ${\text{c}}$ represents the grade of the index; the attractive domain ${\mathbf{I}}_{{{\text{ab}}}}$ can be obtained in Eq. (7).

7 $$I_{{{\text{ab}}}} = \left( {\left| {a_{ij} ,b_{ij} } \right|} \right)$$

When we enlarge the set ${\mathbf{I}}_{{{\text{ab}}}}$ according to the upper and lower bounds of its adjacent intervals, set ${\mathbf{I}}_{{{\text{de}}}}$ is expressed as:

8 $$I_{de} = \left( {\left| {d_{ij} ,e_{ij} } \right|} \right)$$

Based on the relevant references^28^, the level standard ${\mathbf{F}}$ of the index is depicted as:

9 $$F = \left[ {\begin{array}{*{20}c} {{\text{F}}{}_{{{11}}}} & {...} & {{\text{F}}_{{{\text{1j}}}} } \\ {...} & {...} & {...} \\ {{\text{F}}_{{{\text{i1}}}} } & {...} & {{\text{F}}_{{{\text{ij}}}} } \\ \end{array} } \right]$$

where the element $F_{{{\text{ij}}}}$ is depicted as:

10 $$F_{ij} = \frac{c - j}{{c - 1}}a_{ij} + \frac{j - 1}{{c - 1}}b_{ij}$$

When $j = 1$, $F_{{{\text{i}}1}} = a_{i1}$; when $j = {\text{c}}$, then $F_{{{\text{ic}}}} = b_{ic}$; when $j = \frac{{{\text{c}} + 1}}{2}$, then $F_{{{\text{ij}}}} = \frac{{a_{ij} + b_{ij} }}{2}$.

**Figure 5:**
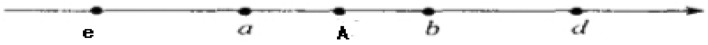
The drawing of position relation.

$X_{0} (a,b)$ is defined as the attractive domain. Namely, when $0 \le D_{F} \left( {\text{u}} \right) \le 1$, ${\mathbf{X}} = [d,e]$ belongs to the upper and lower domain intervals of $X_{0} \left( {X_{0} \subset {\mathbf{X}}} \right)$. Figure 5 show their position relation.

Therefore, their relative membership degree is depicted in Eqs. (11) and (12).

11 $$\left\{ {\begin{array}{*{20}c} {\mu_{F} \left( {\text{u}} \right) = 0.5\left[ {1 + \left( {\frac{x - a}{{F - a}}} \right)^{\beta } } \right];x \in \left[ {a,F} \right]} \\ {\mu_{F} \left( {\text{u}} \right) = 0.5\left[ {1 - \left( {\frac{x - a}{{{\text{d}} - a}}} \right)^{\beta } } \right];x \in \left[ {{\text{d}},a} \right]} \\ \end{array} } \right.$$

12 $$\left\{ {\begin{array}{*{20}c} {\mu_{F} \left( {\text{u}} \right) = 0.5\left[ {1 + \left( {\frac{{x - {\text{b}}}}{F - b}} \right)^{\beta } } \right];x \in \left[ {F,b} \right]} \\ {\mu_{F} \left( {\text{u}} \right) = 0.5\left[ {1 - \left( {\frac{x - b}{{e - b}}} \right)^{\beta } } \right];x \in \left[ {b,{\text{e}}} \right]} \\ \end{array} } \right.$$

### Determining index weights

(1) It is assumed that sample set $X$ can be depicted as follows:

13 $$X = \left[ {\begin{array}{*{20}c} {x_{11} } & {x_{12} } & {...} & {x_{1m} } \\ {x_{21} } & {x_{22} } & {...} & {x_{2m} } \\ {...} & {...} & {...} & {...} \\ {x_{n1} } & {x_{n2} } & {...} & {x_{nm} } \\ \end{array} } \right]$$

(2) Sample set $X_{ij}$ is normalized.

The positive index:

14 $$x_{ij}^{^{\prime}} = \frac{{x_{ij} - \min \left\{ {x_{ij} ,...,x_{nj} } \right\}}}{{\max \left\{ {x_{1j} ,...,x_{nj} } \right\} - \min \left\{ {x_{ij} ,...,x_{nj} } \right\}}}$$

The negative indicator:

15 $$x_{ij}^{^{\prime}} = \frac{{\min \left\{ {x_{ij} ,...,x_{nj} } \right\} - x_{ij} }}{{\max \left\{ {x_{1j} ,...,x_{nj} } \right\} - \min \left\{ {x_{ij} ,...,x_{nj} } \right\}}}$$

where, $i$ is the number of evaluation scheme; $j$ is the number of evaluation index; $x_{ij}$ is the corresponding magnitude.

(3) Determining the proportion of the assessment index.

16 $$b_{ij} = \frac{{x_{ij} }}{{\sum\limits_{i = 1}^{n} {x_{ij} } }}$$

(4) The entropy is calculated in Eq. (17):

17 $$s_{j} = - k\sum\limits_{i = 1}^{n} {b_{ij} \ln \left( {b_{ij} } \right)}$$

(5) The final weight can be depicted in Eq. (18):

18 $$\omega_{j} = \frac{{1 - s_{j} }}{{n - \sum\limits_{j = 1}^{n} {s_{j} } }}$$

### Determining the evaluation grade

According to Eqs. (11), (12) and (18), and in combination with the relevant references^28^, a synthetic membership degree is shown in Eq. (19):

19 $${\text{v}}_{A} \left( {\text{u}} \right)_{j} = \frac{1}{{1 + \left( {\frac{{\sum\limits_{i = 1}^{m} {\left[ {\omega_{i} \left( {1 - \mu_{A} \left( u \right)_{ij} } \right)} \right]^{p} } }}{{\sum\limits_{i = 1}^{m} {\left[ {\omega_{i} \mu_{A} \left( u \right)_{ij} } \right]^{p} } }}} \right)^{\frac{f}{p}} }}$$

Based on Eq. (19), then synthetic membership degree is calculated as:

20 $$V = \left( {{\text{v}}^{^{\prime}} } \right)$$

where

21 $${\text{v}}^{^{\prime}} = \frac{{v_{F} \left( u \right)_{j} }}{{\sum\limits_{j = 1}^{m} {v_{F} \left( u \right)_{j} } }}$$

The evaluation grade $R$ is expressed in Eq. (22).

22 $$R = \left( {1,2,...,{\text{c}}} \right) \bullet V$$

### The calculative step

1. According to the specific data and evaluation standard, the eigenvalue matrix ${\mathbf{X}}$ and classification matrix ${\mathbf{Y}}$ are constructed.
2. The attractive domain ${\mathbf{I}}_{{{\text{ab}}}}$, range matrix ${\mathbf{I}}_{{{\text{de}}}}$ and point value matrix ${\mathbf{F}}$ are constructed.
3. Based on Eqs. (11) and (12), the relative membership degree is calculated.
4. The weights of the rockburst intensity using the proposed model are calculated.
5. The grade eigenvalues $R$ based on the relevant equations are calculated. If $n{ - 0}.5 \le H \le n + 0.5$, then the risk grade is $n$ ($n$ is a nonnegative integer).

## The application of assessment model

### Engineering overview

**Figure 6:**
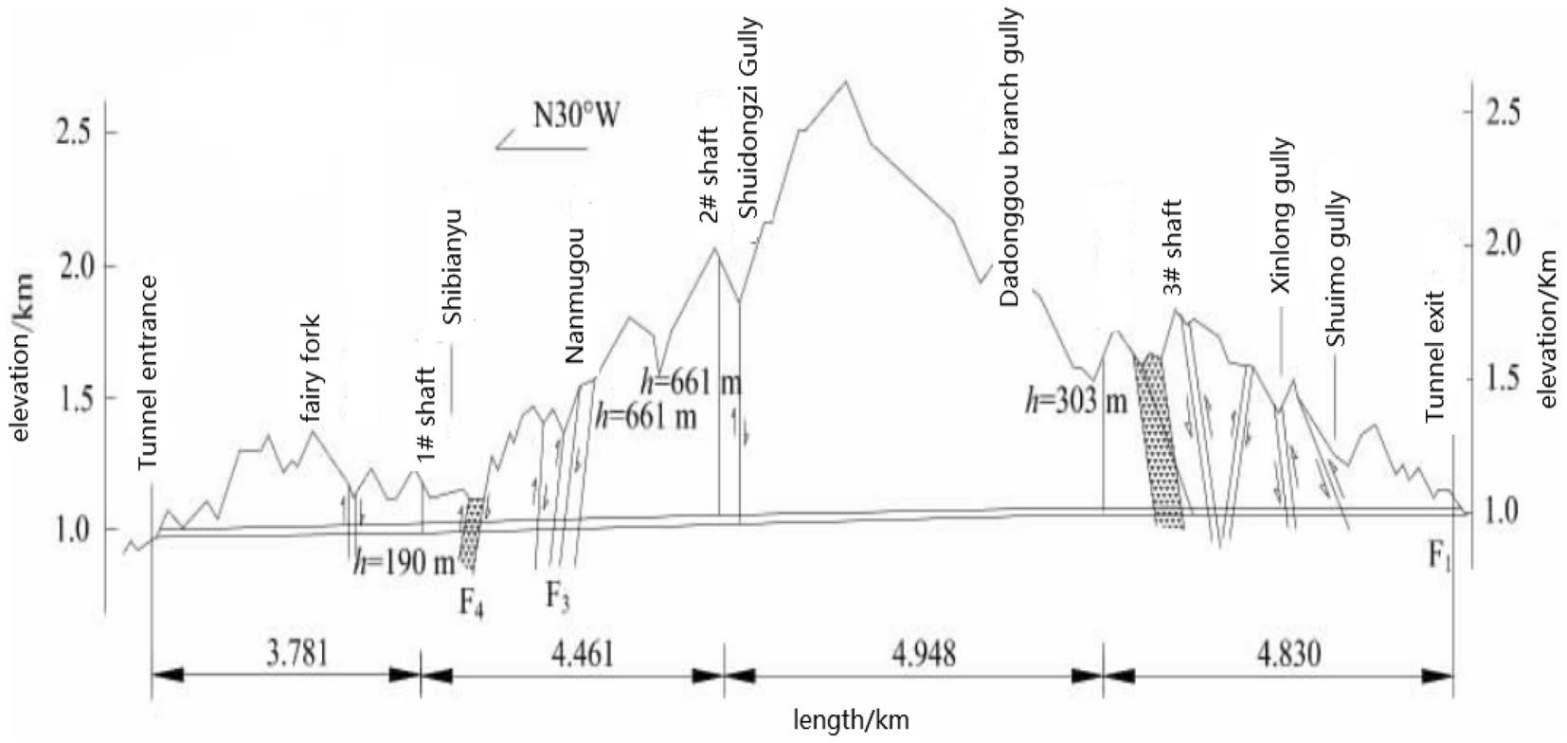
Location of ventilation shafts of tunnel.

**Figure 7:**
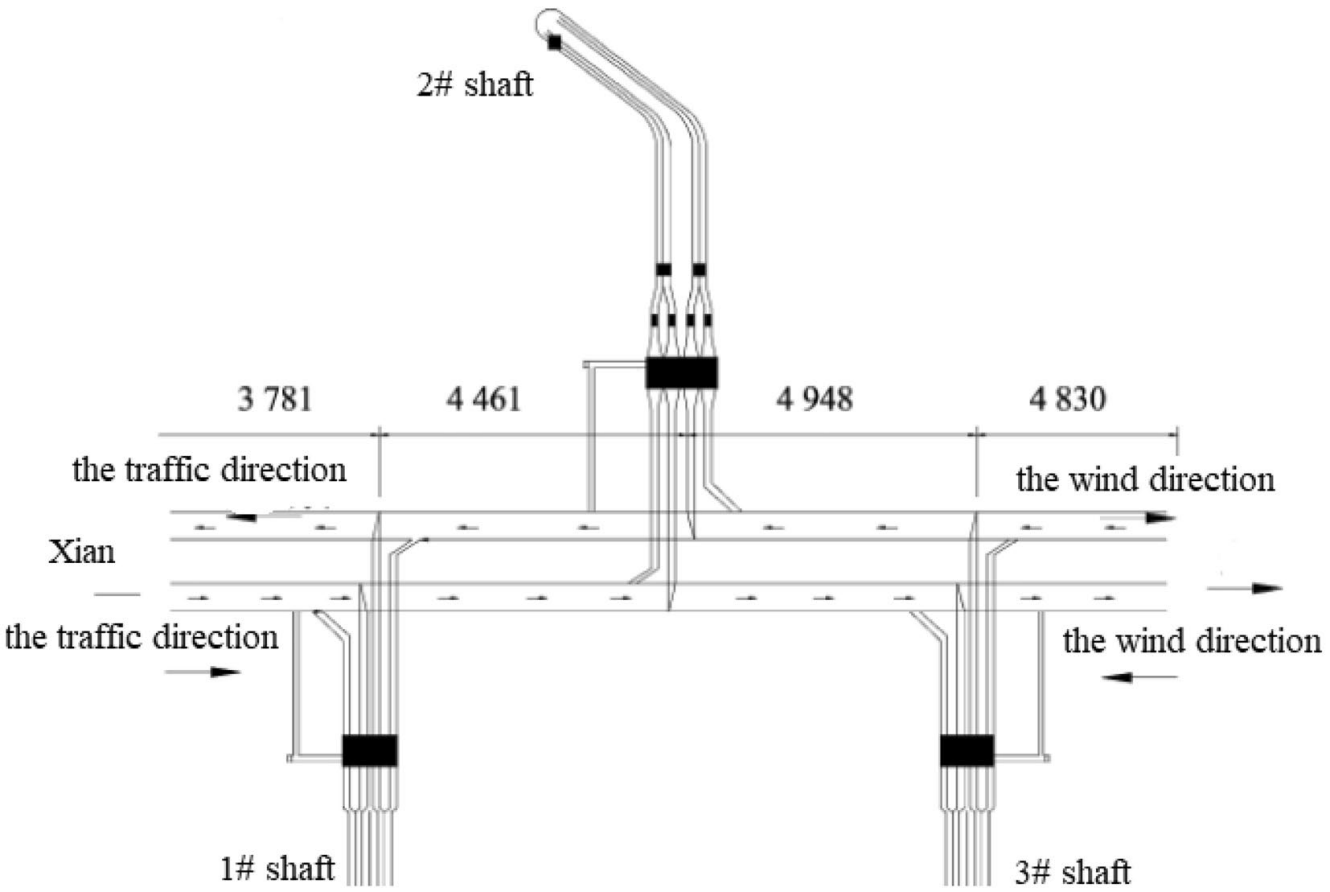
Diagram of ventilation scheme of Zhongnanshan Highway Tunnel.

**Figure 8:**
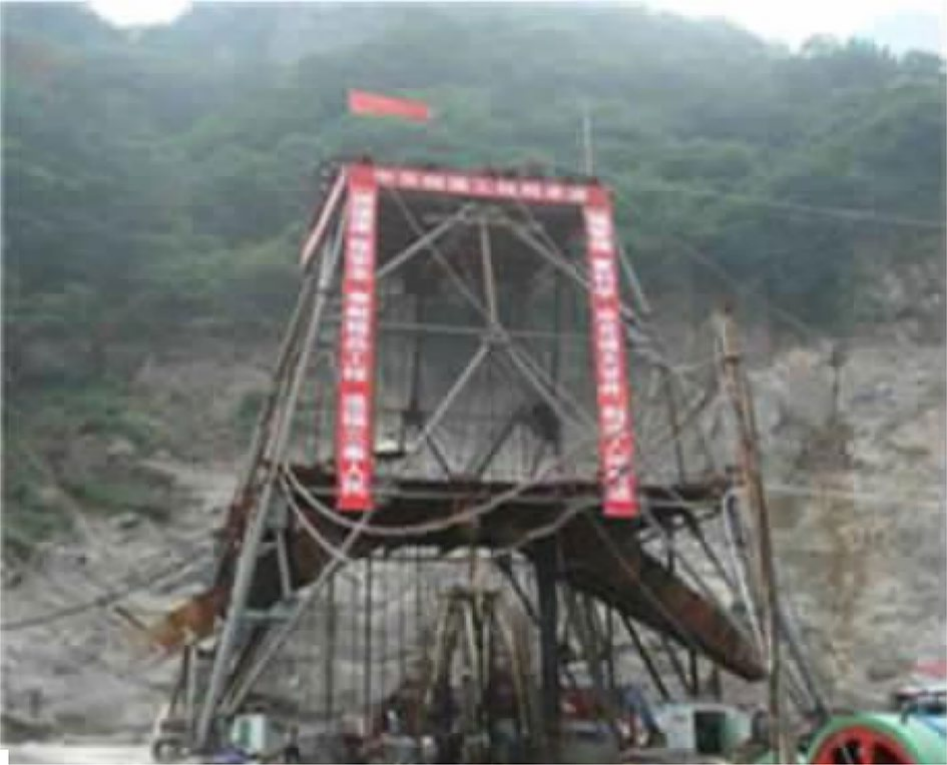
2 # shaft tower.

The total length of the Zhongnanshan Tunnel in the Qinling Mountains, China, is 18.02 km. Three shafts in the projection are applied to improve their environmental conditions by using the method of longitudinal ventilation. It is the deepest ventilation projection of poles in the world, and it is plotted in Figs. 6 and 7. The tunnel is divided into four ventilation sections by three shafts; their lengths are respectively 3.781 km, 4.461 km, 4.948 km, and 4.830 km. The design height of the air Tower of the shaft is selected as 40 m because the height at the lower edge of the vent should be larger than the one at the upper edge. Especially two # shaft is the most extensive of shaft projection in the highway tunnel in China, which is shown in Fig. 8. The inner diameter of the shaft is 11.2 m, the outer diameter of the excavation is 13.32 m, and the ground elevation in the center of the shaft is 1703 m. The depth of the shaft is 661 m. The geological conditions of 2# shaft are listed as follows: the rock stratum at the top 30 m is the Quaternary Holocene slope diluvium, the boulder soil is mixed GNEISS,the lithology belongs to type II surrounding rock; the rock stratum at the bottom is mixed GNEISS, the rock mass is affected slightly by its structure, and it is intact, so the rock mass belongs to type VI surrounding rock. The testing magnitude of maximum horizontal principal stress at the deepest shaft section is 21.04 MPa, the direction is NW28^0^, it belongs to a high-stress level, and the rockburst intensity is high.

### The construction of the evaluation frame

The risk assessment of rockburst intensity has a tremendous influence on the safe construction and the design of the supporting mode. So it has great significance to assess the rockburst intensity.

**Figure 9:**
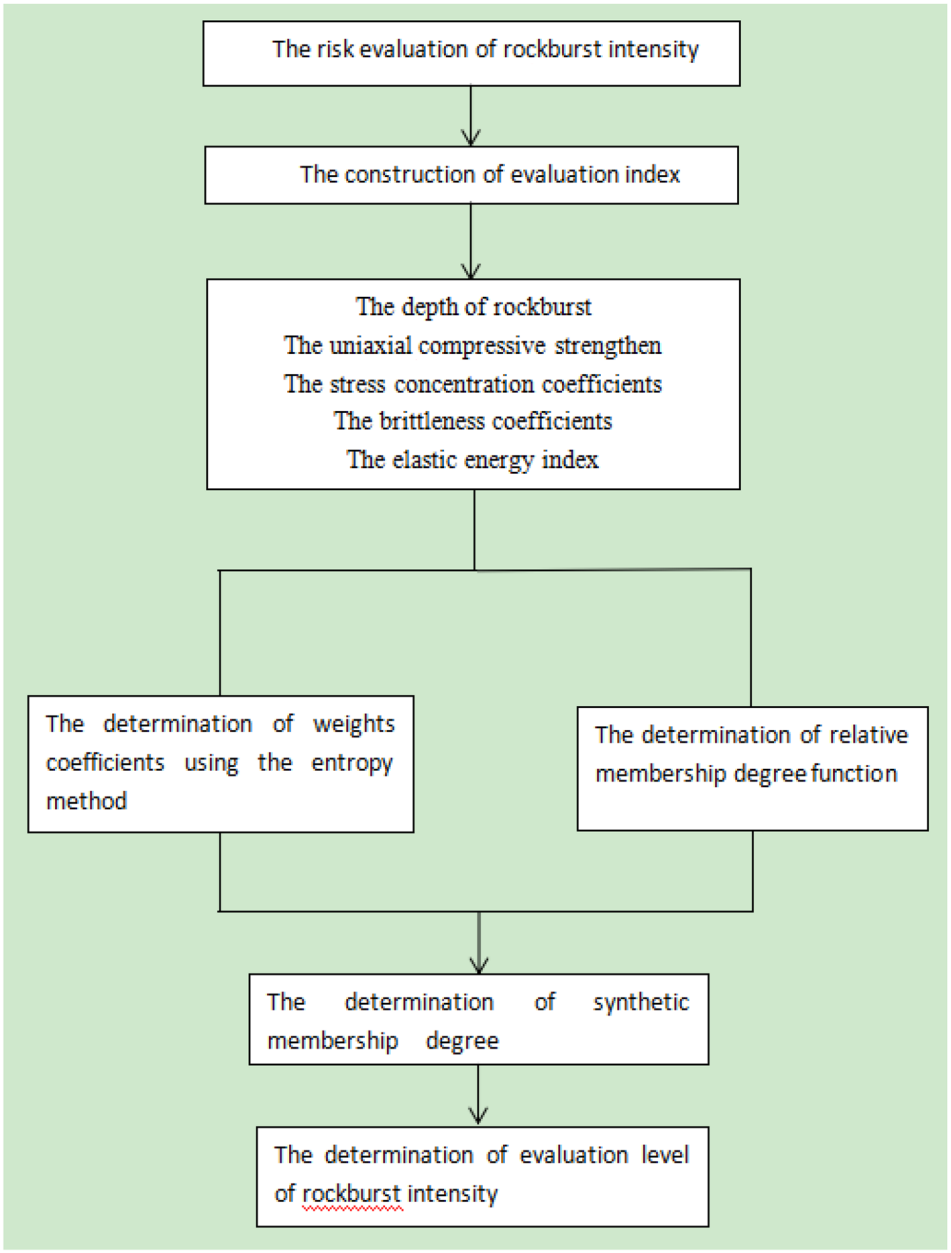
The risk evaluation process of rockburst intensity.

A new evaluation model of rockburst intensity is provided using the variable fuzzy sets theory; its flow chart is plotted in Fig. 9. Firstly, a complete evaluation index system should be constructed before the risk level of rockburst intensity is evaluated. Secondly, Entropy-weight theory is adopted to calculate the weight of each evaluation index. Thirdly, the relative membership degree is defined based on the proposed model. And then, the proposed model can determine the risk level of rockburst intensity.

### Determining the evaluation index

The evaluation index of rockburst should be considered from the internal and outer factors. Usually, internal factors are defined as lithology. The external factors include stress conditions and surrounding rock conditions. To meet with the above factors, the depth of rockburst $D$,the uniaxial compressive strength $\sigma_{{\text{c}}}$, the stress concentration coefficients $SCF$,the brittleness coefficients $B_{1}$ and the elastic energy index $W_{{{\text{et}}}}$ are selected as the evaluation index of rockburst intensity in the paper. According to the relevant references, the five evaluation index can be classified as four levels in Table 1, level I(no rockburst intensity), level II (weak rockburst intensity), level III(medium rockburst intensity), and level IV(strong rockburst intensity). The monitoring value of the assessment index about five cross sections is depicted in Table 2.

**Table 1:** Level classification of rockburst intensity.

Index	Level			
	I	II	III	IV
$D$	< 50	[50 200)	[200 700)	≥ 700
$\sigma_{{\text{c}}}$	< 80	[80 120)	[120 180)	≥ 180
$SCF$	< 0.2	[0.2 0.3)	[0.3 0.55)	≥ 0.55
$B_{1}$	< 10	[10 14)	[14 18)	≥ 18
$W_{{{\text{et}}}}$	< 2	[2 5)	[5 10)	≥ 10

**Table 2:** The monitoring value.

Series number	$D$	$\sigma_{{\text{c}}}$	$SCF$	$B_{1}$	$W_{{{\text{et}}}}$
1# cross section	119	122	0.35	22.68	3.31
2# cross section	283	121	0.72	13.68	9.05
3# cross section	316	124	0.63	14.35	7.74
4# cross section	467	119	0.47	16.5	5.52
5# cross section	659	120	0.52	18.6	4.16

### The determination of risk level about the rockburst intensity

(1) Constructing of the attractive domain, range matrix, and point value matrix.

According to Eq. (7) and in combination with Table 1, the attractive domain $I_{ab}$ is depicted as :

$$I_{{{\text{ab}}}} = \left[ {\begin{array}{*{20}c} {\left[ {\begin{array}{*{20}c} 0 & {50} \\ \end{array} } \right]} & {\left[ {\begin{array}{*{20}c} {50} & {200} \\ \end{array} } \right]} & {\left[ {\begin{array}{*{20}c} {200} & {700} \\ \end{array} } \right]} & {\left[ {\begin{array}{*{20}c} {700} & {1050} \\ \end{array} } \right]} \\ {\left[ {\begin{array}{*{20}c} 0 & {80} \\ \end{array} } \right]} & {\left[ {\begin{array}{*{20}c} {80} & {120} \\ \end{array} } \right]} & {\left[ {\begin{array}{*{20}c} {120} & {180} \\ \end{array} } \right]} & {\left[ {\begin{array}{*{20}c} {180} & {270} \\ \end{array} } \right]} \\ {\left[ {\begin{array}{*{20}c} 0 & {0.2} \\ \end{array} } \right]} & {\left[ {\begin{array}{*{20}c} {0.2} & {0.3} \\ \end{array} } \right]} & {\left[ {\begin{array}{*{20}c} {0.3} & {0.55} \\ \end{array} } \right]} & {\left[ {\begin{array}{*{20}c} {0.55} & {0.825} \\ \end{array} } \right]} \\ {\left[ {\begin{array}{*{20}c} 0 & {10} \\ \end{array} } \right]} & {\left[ {\begin{array}{*{20}c} {10} & {14} \\ \end{array} } \right]} & {\left[ {\begin{array}{*{20}c} {14} & {18} \\ \end{array} } \right]} & {\left[ {\begin{array}{*{20}c} {18} & {27} \\ \end{array} } \right]} \\ {\left[ {\begin{array}{*{20}c} 0 & 2 \\ \end{array} } \right]} & {\left[ {\begin{array}{*{20}c} 2 & 5 \\ \end{array} } \right]} & {\left[ {\begin{array}{*{20}c} 5 & {10} \\ \end{array} } \right]} & {\left[ {\begin{array}{*{20}c} {10} & {15} \\ \end{array} } \right]} \\ \end{array} } \right]$$

Based on Eq. (8), the matrix $I_{{{\text{de}}}}$ can be expressed as:

$$I_{{{\text{de}}}} = \left[ {\begin{array}{*{20}c} {\left[ {\begin{array}{*{20}c} 0 & {200} \\ \end{array} } \right]} & {\left[ {\begin{array}{*{20}c} 0 & {700} \\ \end{array} } \right]} & {\left[ {\begin{array}{*{20}c} {50} & {1050} \\ \end{array} } \right]} & {\left[ {\begin{array}{*{20}c} {200} & {1050} \\ \end{array} } \right]} \\ {\left[ {\begin{array}{*{20}c} 0 & {120} \\ \end{array} } \right]} & {\left[ {\begin{array}{*{20}c} 0 & {180} \\ \end{array} } \right]} & {\left[ {\begin{array}{*{20}c} {80} & {270} \\ \end{array} } \right]} & {\left[ {\begin{array}{*{20}c} {120} & {270} \\ \end{array} } \right]} \\ {\left[ {\begin{array}{*{20}c} 0 & {0.3} \\ \end{array} } \right]} & {\left[ {\begin{array}{*{20}c} 0 & {0.55} \\ \end{array} } \right]} & {\left[ {\begin{array}{*{20}c} {0.2} & {0.825} \\ \end{array} } \right]} & {\left[ {\begin{array}{*{20}c} {0.3} & {0.825} \\ \end{array} } \right]} \\ {\left[ {\begin{array}{*{20}c} 0 & {14} \\ \end{array} } \right]} & {\left[ {\begin{array}{*{20}c} 0 & {18} \\ \end{array} } \right]} & {\left[ {\begin{array}{*{20}c} {10} & {27} \\ \end{array} } \right]} & {\left[ {\begin{array}{*{20}c} {14} & {27} \\ \end{array} } \right]} \\ {\left[ {\begin{array}{*{20}c} 0 & 5 \\ \end{array} } \right]} & {\left[ {\begin{array}{*{20}c} 0 & {10} \\ \end{array} } \right]} & {\left[ {\begin{array}{*{20}c} 2 & {15} \\ \end{array} } \right]} & {\left[ {\begin{array}{*{20}c} 5 & {15} \\ \end{array} } \right]} \\ \end{array} } \right]$$

According to Eqs. (10) and (11), the point value matrix $F$ can be shown as:

$$F = \left[ {\begin{array}{*{20}c} 0 & {100} & {533.3} & {1050} \\ 0 & {93.3} & {160} & {270} \\ 0 & {0.183} & {0.616} & {0.825} \\ 0 & 6 & {21.3} & {27} \\ 0 & {3.33} & {10.67} & {15} \\ \end{array} } \right]$$

(2) The determination of relative membership degree matrix

Based on Table 2, and in combination with Eqs. (11) and (12), we should decide whether the evaluation magnitudes are at the left or right of the point $F$, the data of 1# cross section is adopted as an example when ${\text{i}} = 1$, then $$\left[ {\begin{array}{*{20}c} {\text{a}} & b \\ \end{array} } \right]_{1j}$$, $$\left[ {\begin{array}{*{20}c} {\text{d}} & e \\ \end{array} } \right]_{1j}$$ and $F$ can be respectively depicted as:

$$\left[ {\begin{array}{*{20}c} {\text{a}} & b \\ \end{array} } \right]_{1j} = \left( {\begin{array}{*{20}c} {\left[ {\begin{array}{*{20}c} 0 & {50} \\ \end{array} } \right]} & {\left[ {\begin{array}{*{20}c} {50} & {200} \\ \end{array} } \right]} & {\left[ {\begin{array}{*{20}c} {200} & {700} \\ \end{array} } \right]} & {\left[ {\begin{array}{*{20}c} {700} & {1050} \\ \end{array} } \right]} \\ \end{array} } \right)$$

$$\left[ {\begin{array}{*{20}c} {\text{d}} & e \\ \end{array} } \right]_{1j} = \left( {\begin{array}{*{20}c} {\left[ {\begin{array}{*{20}c} 0 & {200} \\ \end{array} } \right]} & {\left[ {\begin{array}{*{20}c} 0 & {700} \\ \end{array} } \right]} & {\left[ {\begin{array}{*{20}c} {50} & {1050} \\ \end{array} } \right]} & {\left[ {\begin{array}{*{20}c} {200} & {1050} \\ \end{array} } \right]} \\ \end{array} } \right)$$

$$F_{{1{\text{j}}}} = \left[ {\begin{array}{*{20}c} 0 & {100} & {533.3} & {1050} \\ \end{array} } \right]$$

When $x_{1} = 119$, ${\text{a}}_{11} = 0$, ${\text{b}}_{11} = 50$, ${\text{d}}_{11} = 0$, ${\text{e}}_{11} = 200$, $F_{11} = 0$, therefore ${\text{x}}_{1}$ is located in the interval $$\left[ {\begin{array}{*{20}c} {{\text{b}}_{11} } & {e_{11} } \\ \end{array} } \right]$$, so $\mu_{F} \left( {{\text{u}}_{11} } \right) = 0.27$; when ${\text{a}}_{12} = 50$, ${\text{b}}_{12} = 200$, ${\text{d}}_{12} = 0$, ${\text{e}}_{12} = 700$, $F_{12} = 100$, ${\text{x}}_{1}$ is located in the out of interval $$\left[ {\begin{array}{*{20}c} {{\text{F}}_{12} } & {{\text{b}}_{12} } \\ \end{array} } \right]$$, so $\mu_{F} \left( {{\text{u}}_{12} } \right) = 0.905$; when ${\text{a}}_{13} = 200$, ${\text{b}}_{13} = 700$, ${\text{d}}_{13} = 50$,${\text{e}}_{13} = 1050$, $F_{13} = 533.3$, ${\text{x}}_{1}$ is located in the out of interval $$\left[ {\begin{array}{*{20}c} {{\text{d}}_{13} } & {{\text{a}}_{13} } \\ \end{array} } \right]$$, so $\mu_{F} \left( {{\text{u}}_{13} } \right) = 0.23$; when ${\text{a}}_{14} = 700$, ${\text{b}}_{14} = 1050$, ${\text{d}}_{14} = 200$, ${\text{e}}_{14} = 1050$, $F_{14} = 1050$, ${\text{x}}_{1}$ is located in the out of interval $$\left[ {\begin{array}{*{20}c} {{\text{d}}_{13} } & {{\text{a}}_{13} } \\ \end{array} } \right]$$ ,so $\mu_{F} \left( {{\text{u}}_{14} } \right) = 0$;

In the same way, the relative membership degree matrix of 1# cross section can be obtained as follows:

$$\mu_{F} \left( {u_{1j} } \right) = \left[ {\begin{array}{*{20}c} {0.27} & {0.905} & {0.23} & 0 \\ 0 & {0.483} & {0.525} & {0.017} \\ 0 & {0.286} & {0.579} & {0.1} \\ 0 & 0 & {0.24} & {0.76} \\ {0.282} & {0.992} & {0.115} & 0 \\ \end{array} } \right]$$

(3) Determining weight coefficients

Based on Table 2 and in combination with equation (16), Table 3 shows parameter matrix.

**Table 3:** The synthetic parameters of rockburst intensity.

Series number	$D$	$\sigma_{{\text{c}}}$	$SCF$	$B_{1}$	$W_{{{\text{et}}}}$
1# cross section	0.0645	0.2013	0.1301	0.2643	0.1111
2# cross section	0.1535	0.1997	0.2677	0.1594	0.3039
3# cross section	0.1714	0.2046	0.2342	0.1672	0.2599
4# cross section	0.2533	0.1964	0.1747	0.1923	0.1854
5# cross section	0.3574	0.198	0.1933	0.2168	0.1397

Based on Table 3 and Eq. (17), the entropy matrix can be expressed in Table 4.

**Table 4:** The entropy weight matrix.

Index	$D$	$\sigma_{{\text{c}}}$	$SCF$	$B_{1}$	$W_{{{\text{et}}}}$
Index entropy	0.921	0.9999	0.9821	0.9891	0.9592

According to Eq. (18), the entropy matrix can be depicted in Table 5.

**Table 5:** The weight coefficient matrix.

index	$D$	$\sigma_{{\text{c}}}$	$SCF$	$B_{1}$	$W_{{{\text{et}}}}$
Weight coefficients	0.5313	0.0004	0.1205	0.0731	0.2747

(4) Determination of the comprehensive relative membership degree

Based on the Eq. (19), and $\mu_{F} \left( {\mu_{{1{\text{j}}}} } \right)$, the results are calculated in Table 6.

**Table 6:** The comprehensive relative membership vector.

$k$ & ${\text{l}}$	$v_{F} \left( u \right)_{1}$			
$k = 1,l = 1$	0.2209	0.788	0.2413	0.0676
$k = 1,l = 2$	0.2628	0.8174	0.2317	0.0855
$k = 2,l = 1$	0.0744	0.9325	0.0919	0.0052
$k = 2,l = 2$	0.1127	0.9525	0.0833	0.0087

Based on Eqs. (20) and (21), the comprehensive relative membership degree matrix is normalized in Table 7.

**Table 7:** The normalized comprehensive relative membership degree vector.

$k$ & ${\text{l}}$	$v^{\prime }$			
$k = 1,l = 1$	0.1676	0.5979	0.1831	0.0513
$k = 1,l = 2$	0.1881	0.585	0.1658	0.0612
$k = 2,l = 1$	0.0674	0.8446	0.0832	0.0048
$k = 2,l = 2$	0.0974	0.8231	0.072	0.0075

(5) Determining the risk level of the rockburst intensity

According to Eq. (22) and Table 7, the ranking value of 1# cross section can be depicted in Table 8.

**Table 8:** The feature value of 1# cross section.

Sample number	Ranking feature value				Mean value
	$k = 1,l = 1$	$k = 1,l = 2$	$k = 2,l = 1$	$k = 2,l = 2$	
1	2.1181	2.1001	2.0253	1.9896	2.0583

Similarly, the feature value of 1–5# cross section can be shown in Table 9, respectively.

**Table 9:** The assessment values at 5 cross sections.

Sample number	Ranking feature value				Mean value
	$k = 1,l = 1$	$k = 1,l = 2$	$k = 2,l = 1$	$k = 2,l = 2$	
1	2.1181	2.1001	2.0253	1.9896	2.0583
2	3.9657	3.8913	3.9626	3.8621	3.9204
3	3.9296	3.8755	3.9166	3.8496	3.8928
4	2.9527	2.9677	2.9486	2.9628	2.9579
5	3.0201	2.9868	3.1236	3.1323	3.0657

The results that obtained from different methods are contrasted in Table 10.

**Table 10:** The comparison of results from the different models.

Cross section number	Method in the text	The current specification	WOA-KELM
1	II	II	I
2	IV	IV	IV
3	IV	IV	IV
4	III	III	IV
5	III	III	II

The variable fuzzy set model is applied to assess the rockburst intensity. The full results are respectively shown in Tables 9 and 10. It can be found from Table 10 that the risk level of rockburst intensity from the cross section 1 to 5# are different. The rockburst intensity level at 1 # cross section is II; one at the 2 and 3 # cross section is IV; one at the rest cross section is III. It means that the surrounding rocks at 1 # cross section have weak rockburst intensity. The surrounding rocks at the 2 and 3 # cross section have vigorous rockburst intensity. The surrounding rocks at the 4 and 5 # cross section have medium rockburst intensity, so the qualified rate of rockburst intensity in all cross sections arrives at 20%. The rockburst intensity at 1# cross section is weak, however, the rest cross sections is medium or strong, so the necessary consolidation measurement should be taken to prevent the occurrence of rockburst at these cross sections; for example, the rock bolt should be fixed in the surrounding rocks, et al.

Based on the comparative results of the assessment model in Table 10, it can be found the results assessed by the variable fuzzy sets method are entirely consistent with the current specification for five different cross sections. Its accurate rate arrives at 100% in the text method, which is higher than the results from the WOA-KELM (40%)^29^. The conclusion is drawn that it is feasible to estimate the rockburst intensity by using the Entropy weight-variable fuzzy sets model. The conclusion is drawn that it is feasible to estimate the rockburst intensity by using the Entropy weight-variable fuzzy sets model. For example, the uniaxial compressive strength of the 1# cross section is 122, which should belong to level IV according to Table 1. In addition, the degree of membership of the other indices obtained by the variable fuzzy sets model belongs to level II, so the quality level probability of the 1# cross section at level II is more significant than that of grade I, IV, and III. So the rockburst intensity of the 1# cross section only belongs to level III and almost impossibly belongs to levels I, IV, and III. Furthermore, the intensity level of the 2# cross section is more likely to be level IV than that of the 3# cross section because the mean ranking feature value (3.9204) of the 2# cross section for level III is higher than that of the 3# cross section (3.8928). The results obtained using the Entropy weight-variable fuzzy sets model demonstrate the rockburst intensity level accurately and further determine the rockburst intensity ranking for different cross sections at the same level.

## Discussion

### Comparison with existing studies

The variable fuzzy sets method is provided to assess the risk level of rockburst intensity, and the results are promising. However, due to lack of information, the uncertain human mind, and time complexity, the decision experts (DEs) cannot provide accurate results for the subjective methods, like the Grey fuzzy Comprehensive Evaluation method, level-based weight assessment (LBWA), et al. While, the proposed model conquer this concern. It not only considers the unreliability or reliability of the problem but also solves some degrees of uncertainty and ambiguity of the datum. So it has significant advantages over these subjective ones.

### The advantages and limitations of the proposed model

In comparison with the traditional models, the advantages of the variable fuzzy sets theory are analyzed as follows:

1. The variable fuzzy sets method can accurately demonstrate the risk degree of rockburst intensity using the eigenvalue of level H.
2. Interval-oriented evaluation, not point assessment, is applied in the proposed model, so the reliability of evaluation outcomes is enhanced, and the quality state of rockburst intensity can be discovered with effect.

## Conclusions

Considering rocks' uniaxial compressive strength $\sigma_{c}$, the depth of rockburst $D$, the stress concentration coefficients $SCF$,the brittleness coefficients $B_{1}$, and the elastic energy index $W_{{{\text{et}}}}$, a new assessment method is introduced in this paper to assess the rockburst intensity level. The relative membership degree matrix of the assessment sample is determined at first. Then the weighting coefficients are calculated by using the entropy weighting method. Finally, the rockburst intensity level is determined by using the mean ranking feature value.

The proposed method is applied to assess the rockburst intensity level. Finally, its result is compared with that of the current specifications and the WOA-KELM theory; it is found the results obtained based on the variable fuzzy sets method are entirely consistent with the current specification; its accuracy arrives at 100%. The qualified rate of surrounding rock quality in all cross sections arrives at 20%. In other words, except for 1# cross section, for other cross sections, necessary measures should be adopted to consolidate the surrounding rock. And the results obtained by using the Entropy weight-variable fuzzy sets model demonstrate the rockburst intensity level not only accurately but also further determine the level ranking of rockburst intensity for different cross sections at the same level. The findings of the proposed model provide an alternate way to assess the risk level of rockburst intensity and improve the evaluation accuracy in the future.

## Data Availability

The data used to support the findings of this study are available from the corresponding author upon request.

## References

[1] Zhou XP, Cheng H, Feng YF (2014). An experimental study of crack coalescence behaviour in rock-like materials containing multiple flaws under uniaxial compression. Rock Mech. Rock Eng..

[2] Gu XB, Ma Y, Wu QH, Ji XJ, Bai H (2021). The risk assessment of landslide hazards in Shiwangmiao based on intuitionistic fuzzy sets-Topsis model. Nat. Hazards.

[3] Gu XB, Wu QH (2019). Seismic stability analysis of waterfront rock slopes using the modified pseudodynamic method. Geotech. Geol. Eng..

[4] Zhou XP, Gu XB, Qian QH (2016). Seismic bearing capacity of shallow foundations resting on rock masses subjected to seismic loads. KSCE J. Civ. Eng..

[5] Zhou KP, Lin Y, Hong-wei D, Jie-lin L, Chuan L (2016). Prediction of rock burst classification using cloud model with entropy weight. Trans. Nonferr. Metals Soc. China.

[6] Li LY (2020). Rock Burst Rating Based on Improved Catastrophe Progression Method.

[7] Gu XB, Wu QH (2016). The application of nonordinary, state-based peridynamic theory on the damage process of the Advances in Materials Science and Engineering rock-like materials. Math. Probl. Eng..

[8] Xue Y, Cao ZZ, Shen WL (2019). Destabilization and energy characteristics of coal pillar in roadway driving along gob based on rock burst risk assessment. R. Soc. Open Sci..

[9] Gu XB, Shao JL, Wu ST, Wu QH, Bai H (2021). The risk assessment of Debris flow hazards in Zhouqu based on the projection pursuit classification model. Geotech. Geol. Eng..

[10] Gu XB, Wu ST, Ji XJ, Zhu YH (2021). The risk assessment of debris flow hazards in Banshanmen gully based on the entropy weightnormal cloud method. Adv. Civ. Eng..

[11] Zhang Y, Xu Z, Chu K (2021). Research on operation risk assessment of Zhongnanshan tunnel based on fuzzy comprehensive evaluation method. J. Catastrophol..

[12] Wang Y, Shang Y, Sun H, Yan X (2010). Study of prediction of rockburst intensity based on efficacy coefficient method. Rock Soil Mech..

[13] Zhou XP, Zhang YX, Ha QL, Zhu KS (2008). Micromechanical modelling of the complete stress–strain relationship for crack weakened rock subjected to compressive loading. Rock Mech. Rock Eng..

[14] Gu X-B, Wang L, Wu Q-H (2022). The risk assessment of debris flow in the Duba river watershed using intuitionistic fuzzy sets: TOPSIS model. Math. Probl. Eng..

[15] Zhou R, Wei Z, Zhang Y, Zhang S (2017). A prediction of reference crop evapotranspiration based on generalized regression neural network and particle swarm optimization algorithm. China Rural Water Hydropower.

[16] Zhai Q, Gu W, Zhao Y (2021). Risk assessment of gas disaster in tunnel construction based on unascertained measurement theory. J. Railw. Sci. Eng..

[17] Chen J, Shou Y, Zhou X (2022). Implementation of the novel perfectly matched layer element for elastodynamic problems in time-domain finite element method. Soil Dyn. Earthq. Eng..

[18] Zhou J, Li X, Shi X (2012). Long-term prediction model of rockburst in underground openings using heuristic algorithms and support vector machines. Saf. Sci..

[19] Zhang J, He C (2021). Discussion on the applicability of XGBoost algorithm based on cross validation in prediction of rockburst intensity classification. Tunn. Constr..

[20] Liu X, Yang W, Zhang X (2021). Research on the multidimensional cloud model based on weighted fusion rock burst prediction. Chin. Min. Mag..

[21] Liang W (2021). Probability estimates of short-term rockburst risk with ensemble classifiers. Rock Mech. Rock Eng..

[22] Zhao G, Liu L (2019). PCA-OPF Model for rock burst prediction. Min. Metall. Eng..

[23] Gu X-B, Wu Q-H, Ma Y (2022). Risk assessment of the rockburst intensity in a hydraulic tunnel using an intuitionistic fuzzy sets-TOPSIS model. Adv. Mater. Sci. Eng..

[24] Yu, G. & Shouyu, C. [American Society of Civil Engineers GeoShanghai International Conference 2006 - Shanghai, China (June 6–8, 2006)] Soil and Rock Behavior and Modeling—Application of Variable Fuzzy Sets in Classified Prediction of Rockburst. 112–118.10.1061/40862(194)14 (2006)

[25] Wang, M., Jin, J., & Li, L. SPA-VFS model for the prediction of rockburst. In *2008 Fifth International Conference on Fuzzy Systems and Knowledge Discovery*, vol. 5, 34–38 (IEEE, 2008).

[26] Wang H, Nie L, Xu Y, Lv Y, He Y, Du C, Zhang T, Wang Y (2019). Comprehensive prediction and discriminant model for rockburst intensity based on improved variable fuzzy sets approach. Appl. Sci..

[27] Wang WC, Xu DM, Lei KW, Lei GJ (2014). Assessment of river water quality based on theory of variable fuzzy sets and fuzzy binary comparison method. Water Resour. Manag..

[28] Wang YK, Sheng D, Wang D, Ma H, Wu J, Xu F (2014). Variable fuzzy set theory to assess water quality of the meiliang bay in taihu lake basin. Water Resour. Manag..

[29] Zhao, S. *Classified prediction model of rockburst using KPCA-WOA-KELM*. PH.D. (Hebei University of Engineering, Handan, Hebei province, China, **2021).**

